# Calcium-Activated Potassium Current Modulates Ventricular Repolarization in Chronic Heart Failure

**DOI:** 10.1371/journal.pone.0108824

**Published:** 2014-10-01

**Authors:** Ingrid M. Bonilla, Victor P. Long, Pedro Vargas-Pinto, Patrick Wright, Andriy Belevych, Qing Lou, Kent Mowrey, Jae Yoo, Philip F. Binkley, Vadim V. Fedorov, Sandor Györke, Paulus M. L. Janssen, Ahmet Kilic, Peter J. Mohler, Cynthia A. Carnes

**Affiliations:** 1 College of Pharmacy, The Ohio State University, Columbus, Ohio, United States of America; 2 Dorothy M. Davis Heart and Lung Research Institute, The Ohio State University Wexner Medical Center, Columbus, Ohio, United States of America; 3 College of Veterinary Medicine, The Ohio State University, Columbus, Ohio, United States of America; 4 St Jude Medical, Sylmar, California, United States of America; University of Oxford, United Kingdom

## Abstract

The role of I_KCa_ in cardiac repolarization remains controversial and varies across species. The relevance of the current as a therapeutic target is therefore undefined. We examined the cellular electrophysiologic effects of I_KCa_ blockade in controls, chronic heart failure (HF) and HF with sustained atrial fibrillation. We used perforated patch action potential recordings to maintain intrinsic calcium cycling. The I_KCa_ blocker (apamin 100 nM) was used to examine the role of the current in atrial and ventricular myocytes. A canine tachypacing induced model of HF (1 and 4 months, n = 5 per group) was used, and compared to a group of 4 month HF with 6 weeks of superimposed atrial fibrillation (n = 7). A group of age-matched canine controls were used (n = 8). Human atrial and ventricular myocytes were isolated from explanted end-stage failing hearts which were obtained from transplant recipients, and studied in parallel. Atrial myocyte action potentials were unchanged by I_KCa_ blockade in all of the groups studied. I_KCa_ blockade did not affect ventricular myocyte repolarization in controls. HF caused prolongation of ventricular myocyte action potential repolarization. I_KCa_ blockade caused further prolongation of ventricular repolarization in HF and also caused repolarization instability and early afterdepolarizations. SK2 and SK3 expression in the atria and SK3 in the ventricle were increased in canine heart failure. We conclude that during HF, I_KCa_ blockade in ventricular myocytes results in cellular arrhythmias. Furthermore, our data suggest an important role for I_KCa_ in the maintenance of ventricular repolarization stability during chronic heart failure. Our findings suggest that novel antiarrhythmic therapies should have safety and efficacy evaluated in both atria and ventricles.

## Introduction

Heart failure (HF) is a chronic disease that develops over months to years, and is defined by insufficient cardiac output to meet the physiologic and metabolic needs of the body. Atrial fibrillation (AF) and HF are common coexisting disease states, and HF results in a 4.5 to 5.9 fold increase in the risk of developing AF. [1] Moreover, in patients with HF, the development of AF significantly increases the risk of death. [2] Thus, identifying and elucidating pharmacological targets to treat AF may significantly reduce mortality and morbidity in HF.

Small-conductance Ca^2+^- activated K^+^ (SK) channels are expressed in multiple tissues such as skeletal and smooth muscle, the central and peripheral nervous system and the heart.[3]–[5] Cardiac myocytes express SK1, SK2 and SK3 gene products. [6] SK- encoded current is voltage-independent and activated by intracellular calcium. [7] All three members of the SK family have similar calcium sensitivity for activation (0.6–0.7 µM) [8]. SK-encoded current is blocked by apamin, a constituent of bee venom, which appears to be highly selective for I_KCa_. [7], [9], [10].

I_KCa_, the potassium current conducted by SK channels, contributes to repolarization, [3], [11] but the importance of I_KCa_ in repolarization remains poorly elucidated. For example, ventricular I_KCa_ shortens repolarization and promotes peri-infarct arrhythmias in rats. [12] Conversely, blockade of I_KCa_ promotes ventricular arrhythmias in human HF and a non-ischemic rabbit HF model, suggesting a protective role for I_KCa_. [13], [14] The contribution of I_KCa_ to *atrial* repolarization is also unclear as some reports demonstrate that I_KCa_ is proarrhythmic while others suggest it is protective. [15], [16].

We measured the impact of I_KCa_ block on action potentials in intact myocytes using perforated patch recordings to maintain intrinsic Ca^2+^cycling. We utilized a well-validated canine model that emulates all key features of human HF including chamber dilatation, impaired contractility, impaired functional capacity, repolarization abnormalities, dysregulated myocyte calcium handling, increased predisposition to AF and increased myocardial fibrosis. [17], [18] Complementary experiments were conducted in end-stage human HF. The role of I_KCa_ in AF was evaluated in atrial myocytes from a canine model of chronic HF with sustained AF.

## Methods

### Heart failure and atrial fibrillation animal models

All animal procedures conformed to the Guide for the Care and Use of Laboratory Animals of the National Institutes of Health, and were approved by the Ohio State University Institutional Animal Care and Use Committee (Protocol: 2010A00000103-R1). Canine heart failure was induced by right ventricular (RV) tachypacing as previously described. [19] Animals were assigned to one or four months of RV tachypacing to induce heart failure.

Atrial fibrillation was induced in dogs with HF using a customized pacemaker (St Jude Medical, Sylmar, CA). One pacing lead was implanted in the right atria (RA) and the second lead was implanted in the RV, with HF induced as previously described. [20] After 10 weeks of RV tachypacing, RA tachypacing was initiated, with the RA stimulated at 10 Hz for 60 seconds, followed by a 10 second pause for automated interrogation of atrial rhythm. This cycle of RA tachypacing was repeated every 70 seconds until AF was detected. Subsequent detection of normal atrial rhythm resulted in resumption of the atrial tachypacing. The total HF duration in the HF+AF group was 4 months. Ventricular pacing was stopped during atrial stimulation, and the ventricular rate was 150–200 BPM during atrial pacing or AF. Serial echocardiograms and ECGs were performed as previously reported. [17], [21] Serial pacemaker interrogations were used to monitor cardiac rhythm.

### Myocyte Isolation and Tissue Collection

On the day of the terminal procedure, the dogs were anesthetized with pentobarbital sodium (50 mg/kg intravenously; Nembutal, Abbott Laboratories). The heart was rapidly removed and perfused with cold cardioplegia solution containing the following (mM): NaCl 110, CaCl_2_ 1.2, KCl 16, MgCl_2_ 16 and NaHCO_3_ 10. Cannulation of the left circumflex artery was used to perfuse left atria and ventricle following removal of the right atrium and right ventricle, as previously described. [22] Adjacent tissue samples were collected and snap frozen for protein analyses. Tyrode’s solution (mM) containing NaCl 130, KCl 5.4, MgCl_2_ 3.5, NaH_2_PO_4_ 0.5, Glucose 10, HEPES 5 and taurine 20, was used as the initial perfusate. During the cell isolation process the heart was perfused with three different solutions (36°C). The heart was initially perfused for 10 minutes with Tyrode’s solution with 0.1 mM EGTA; this was followed by perfusion with Tyrode’s solution containing 0.3 mM Calcium, 0.12 mg/ml of Trypsin Inhibitor (NIBCO) and 1.33 mg/ml of collagenase (Type II, Worthington) for a maximum of 45 minutes. Then following enzymatic digestion, the heart was perfused with normal Tyrode’s solution for five minutes to remove residual enzyme. Subsequently, left ventricular mid-myocardial and left atrial myocytes were obtained through secondary digestion, as previously described. [22] After secondary digestion the cells were re-suspended in incubation buffer. [23] This isolation procedure typically yields 70–90% and 40–60% rod shaped ventricular and atrial myocytes, respectively. All myocyte electrophysiology experiments were conducted within 10 hours of isolation.

In parallel experiments, failing human cardiac tissue was obtained after written informed consent with the documentation of consent securely stored as approved by the Institutional Review Board approval of The Ohio State University (IRB 2008H0113 and IRB 2012H0197), in accordance with the 1964 Declaration of Helsinki and its later amendments. Additional human cardiac tissue was obtained from the Lifeline of Ohio Organ Procurement program (http://lifelineofohio.org). For these tissues, the Institutional Review Board waived the need for consent and these tissues were used according to the Ohio State University guidelines regarding the use of data and/or specimens.

Left ventricular mid-myocardial and left atrial appendage myocytes were isolated and adjacent tissues were collected from explanted end-stage failing hearts (n = 6; obtained from the Ohio State University Wexner Medical Center transplant program). After cannulation of a superficial coronary artery to perfuse the left atrium and/or left ventricle, the methods for myocyte isolation were as described for canine samples above. Left ventricular mid myocardium and left atrial appendege tissues were collected from non-failing heart for Western blotting purposes (n = 8 obtained from Lifeline of Ohio). Non-HF status was confirmed in these tissues by lack of CaMKII pS286 hyperphosphorylation.

### Action Potential (AP) Measurements

Amphotericin-B perforated patch clamp techniques with a bath temperature of 36±0.5°C were used. The myocytes were placed in a laminin-coated cell chamber (Cell Microcontrols, Norfolk, VA) and superfused (∼1 mL/min) with bath solution containing (mM): 135 NaCl, 5 MgCl_2_, 5 KCl, 10 glucose, 1.8 CaCl_2_, and 5 HEPES with pH adjusted to 7.40 with NaOH. Borosilicate glass micropipettes with tip resistance of 1.5–3 MΩ, were filled with pipette solution containing the following (mM): 100 K-aspartate, 40 KCl, 5 MgCl, 5 EGTA, 5 HEPES, pH adjusted to 7.2 with KOH.

APs were recorded in a train of 25 traces at 0.5, 1 and 2 Hz at baseline and after apamin perfusion. The average of the last 10 traces (i.e. from trace 16–25) was used to calculate the action potential duration (APD). APD was calculated at 50 and 90 percent of repolarization (APD_50_ and APD_90_).

To evaluate repolarization instability, beat to beat variability (BTBV) of APD_90_ was assessed as the standard deviation of the APD_90_, as previously described. [24], [25] Early afterdepolarization (EAD) propensity was assessed as the percentage of cells exhibiting EADs. Recordings exhibiting EADs were excluded from APD and BTBV measurements.

Data collection was done at baseline and after superfusion with the I_KCa_ blocker apamin (100 nM), a concentration known to block SK1, SK2 and SK3 encoded-current.[26]–[28] An Axopatch 200A amplifier with Digidata 1440A (Molecular devices, Sunnyvale, CA) and Clampex 10.2 software was used for data acquisition. At the initiation of each recording the resting potential was examined. For canine cells, atrial cells with a resting membrane potential of ≥ −55 mV were recorded; for ventricular cells those with a resting membrane potential of ≥ −70 mV were recorded. For the human cells, every cell with complete baseline and apamin-treatment data was included. One apamin-treated ventricular canine cell action potential recording was excluded as an outlier.

### Calcium transient Measurements

Calcium transients were recorded using Ca^2+^ sensitive dye Fluo-4AM (10 µM) and an Olympus Fluoview 1000 confocal microscope in line scan mode. Myocytes were loaded with dye for 25 minutes at room temperature. Fluo-4 was excited with a 488 nm argon laser and fluorescence collected at wavelength 500–600 nm. Myocytes were paced by extracellular stimulation at 1 Hz with platinum electrodes. External solution contained (mM): 140 NaCl, 5.4 KCl, 2 CaCl_2_, 0.5 MgCl_2_, 10 HEPES and 5.6 glucose (pH 7.3).

### Immunoblots

Following protein quantification, tissue lysates were analyzed on Mini-PROTEAN tetra cell (BioRad) on a 4–15% precast TGX gel (BioRad) in Tris/Glycine/SDS Buffer (BioRad). Gels were transferred to a nitrocellulose membrane using the Mini-PROTEAN tetra cell (BioRad) in Tris/Glycine buffer with 20% methanol (v/v, BioRad). Membranes were blocked for 1 hour at room temperature using a 3% BSA solution and incubated with primary antibody overnight at 4°C. Antibodies included: SK2 (Alomone, Santa Cruz), SK3 (Alomone, Santa Cruz), GAPDH (Fitzgerald), and actin (Sigma). Secondary antibodies included donkey anti-mouse-HRP and donkey anti-rabbit-HRP (Jackson Laboratories). Densitometry was performed using Image lab software and all data was normalized to GAPDH or actin levels present in each sample.

### Data Analysis

Cellular electrophysiology and Ca^2+^ imaging data were analyzed using Clampfit 10.3 software (Axon Instruments) and Origin 9.0 software (OriginLab, Northampton, MA, USA). APD data was examined for outliers by application of the Grubb’s test, which rejected one control ventricular myocyte (GraphPad). All APD paired data were compared by paired student t-test. Unpaired data and comparisons between groups were analyzed by one-way ANOVA with post hoc least significant difference testing. Differences in EADs incidence were tested with Pearson’s Chi-Square test. For protein experiments, differences were assessed with a paired Student’s *t* test (2-tailed) or ANOVA, as appropriate, for continuous data. The Bonferroni test was used for post-hoc testing. All data are presented as mean ± SE and p<0.05 was the criterion for statistical significance for all comparisons.

### Chemicals

All chemicals were purchased from Sigma-Aldrich (St. Louis, MO, USA) and Fisher Scientific (Pittsburg, PA, USA), unless otherwise noted. All buffers and solutions were prepared daily.

## Results

### In vivo cardiac remodeling

**Figure 1:**
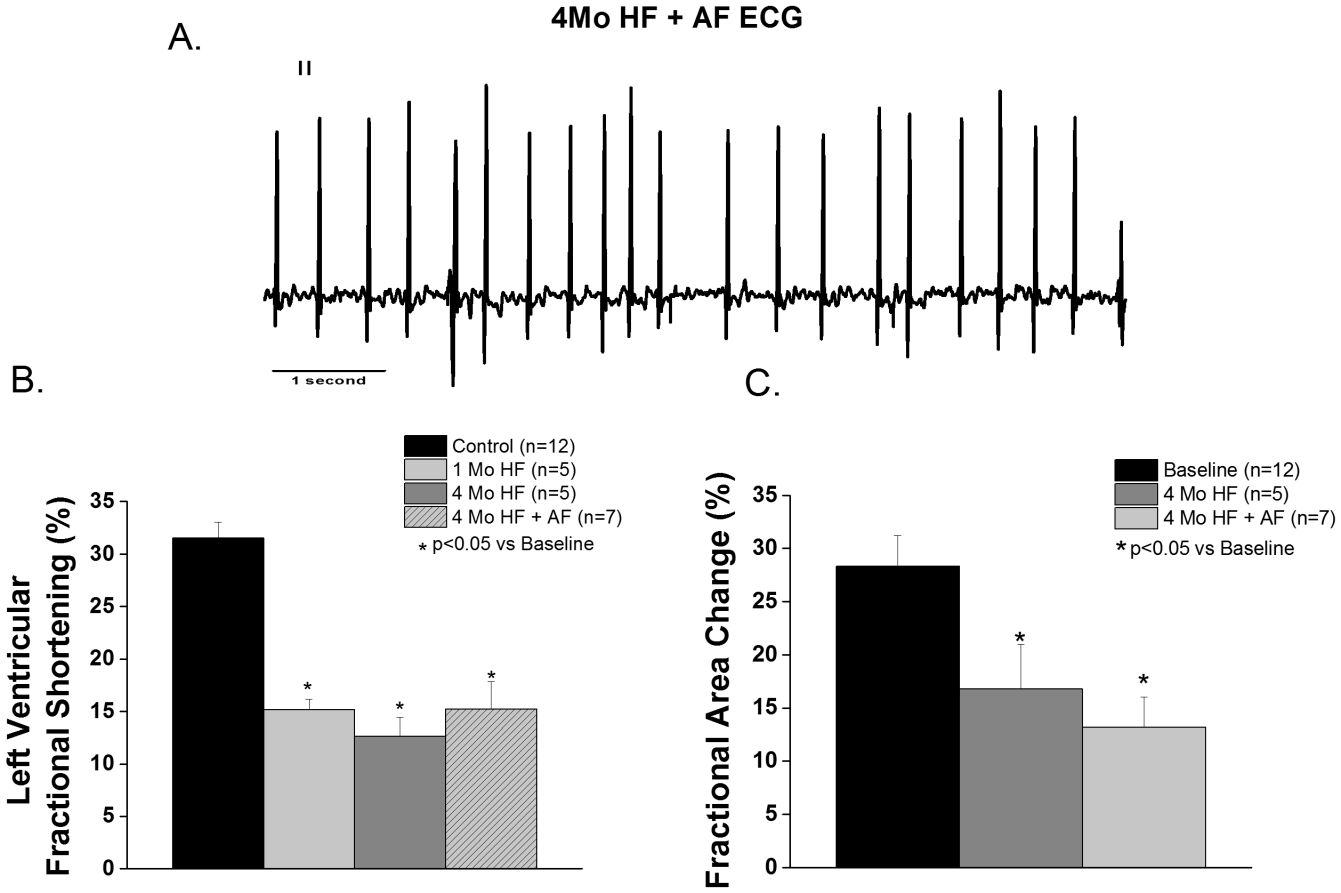
In vivo data from 1 month (1 Mo), 4 month (4 Mo), and 4 month HF with sustained AF (4 Mo HF+AF) canine groups. **A.** Representative ECG recording from a 4 month HF+AF dog showing the absence of P waves and irregularly irregular QRS complexes characteristic of AF. **B.** LVFS was similarly decreased in the 1 month HF, 4 month HF and the 4 month HF+AF groups compared to baseline. (p<0.05 vs baseline). **C**. Atrial contractility was decreased in 4 month HF and 4 month HF+AF groups compared to baseline. (p<0.05, N = 5–7 per group).

Left ventricular fractional shortening (LVFS) was similarly reduced in the 1 month HF, 4 month HF and 4 month HF+AF groups (Figure 1B), consistent with HF. Electrocardiograms (ECGs) in all canines assigned to the HF+ AF group demonstrated sustained atrial tachyarrhythmias, evident as the absence of P waves and the irregularly irregular ventricular rate characteristic of AF (Figure 1A). Additionally, atrial contractility, measured as left atrial fractional area change (FAC), was significantly reduced in both the 4 month HF and 4 month HF+AF groups compared to baseline (p<0.05) as shown in Figure 1C. Notably, the presence of sustained AF did not cause a further decrement in atrial contractility compared to HF alone.

### I_KCa_ inhibition in control ventricular myocytes

**Figure 2:**
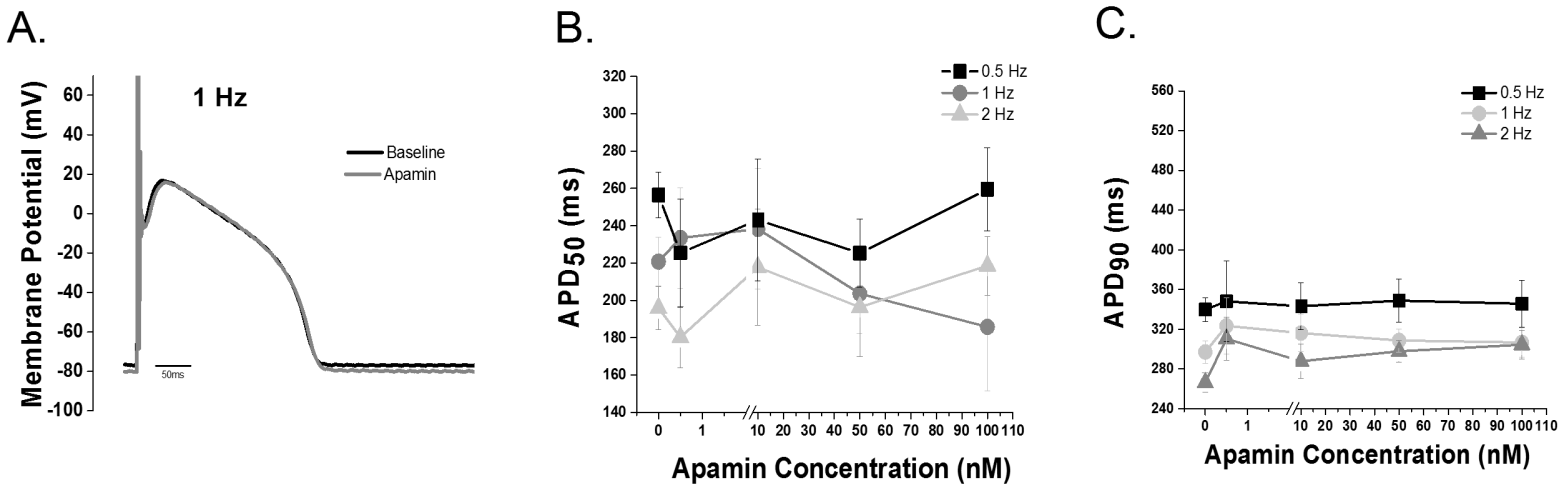
I_KCa_ inhibition does not alter repolarization in control ventricular cells. **A**. Representative action potential tracing before and after 100 nM apamin recorded at 1 Hz. **B.** APD_50_ and **C.** APD_90_ dose response data (0–100 nM apamin) recorded at stimulation rates of 0.5, 1 and 2 Hz. (p = NS, n = 5–11 cells per group; 8 animals). The Grubb’s test for outlier data was applied and one cell was rejected and is not included in the summary data.

Action potentials before and after apamin treatment were recorded from control canine ventricular myocytes. Varying apamin concentrations (0.5–100 nM) were tested in order to generate a concentration response curve. Apamin did not alter APD_50_ or APD_90_ in control ventricular myocytes (Figure 2A).

### I_KCa_ inhibition and SK expression in failing ventricle

#### Canine

**Figure 3:**
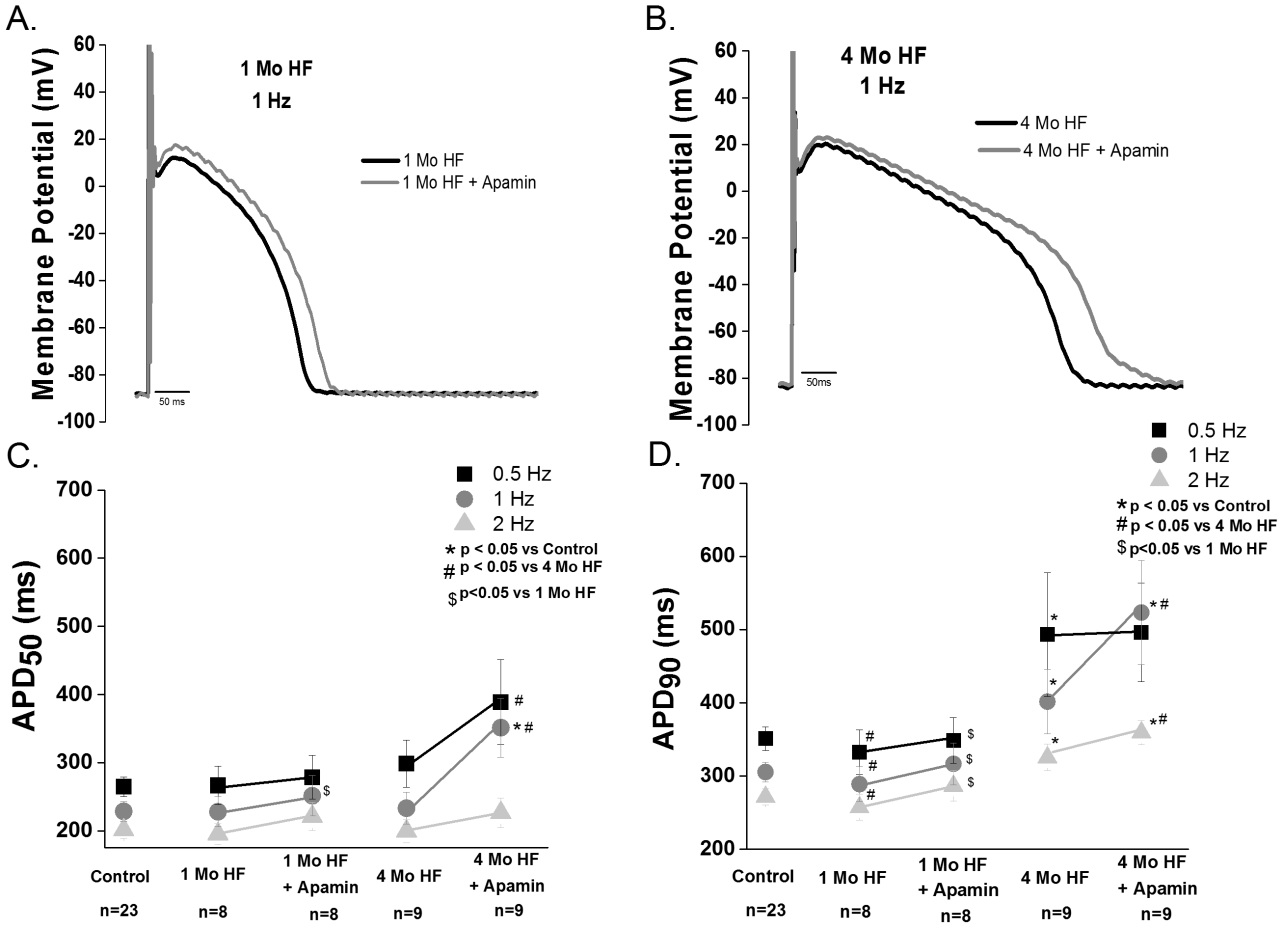
Apamin modulates ventricular repolarization during HF. Representative action potential of a 1 month (**A)** HF and 4 month HF(**B)** ventricular cell before and after apamin superfusion. **C.** Summary data of APD_50_ in control,1 month and 4 month HF before and after apamin treatment. No difference between 1 month HF, 4 month HF and control is observed (2–8 animals per group). Apamin treatment of 1 month HF cells causes a prolongation only at 2 Hz(p<0.05), likewise apamin treatment of 4 month HF cells causes a prolongation at 0.5 Hz (p<0.05) and 1 Hz (p<0.05). **D**. Apamin prolongs APD_90_ in both 1 and 4 month HF (p<0.05).

We observed no HF-induced difference in APD_50_ or APD_90_ in the 1 month HF myocytes compared with control myocytes. However, apamin (100 nM) caused a significant prolongation of the APD_90_ in one month HF (p<0.05; Figure 3 A–D). In contrast to 1 month HF, 4 month HF significantly increased APD_90_ relative to control ventricular myocytes (p<0.05 vs control). Furthermore, when I_KCa_ was blocked in 4 month HF ventricular myocytes (100 nM apamin), there was a significant prolongation of the APD_50_ at lower rates (i.e. p<0.05 vs control at 0.5 and 1 Hz) and a further prolongation in the APD_90_ (p<0.05 vs control and baseline 4 month HF).

**Figure 4:**
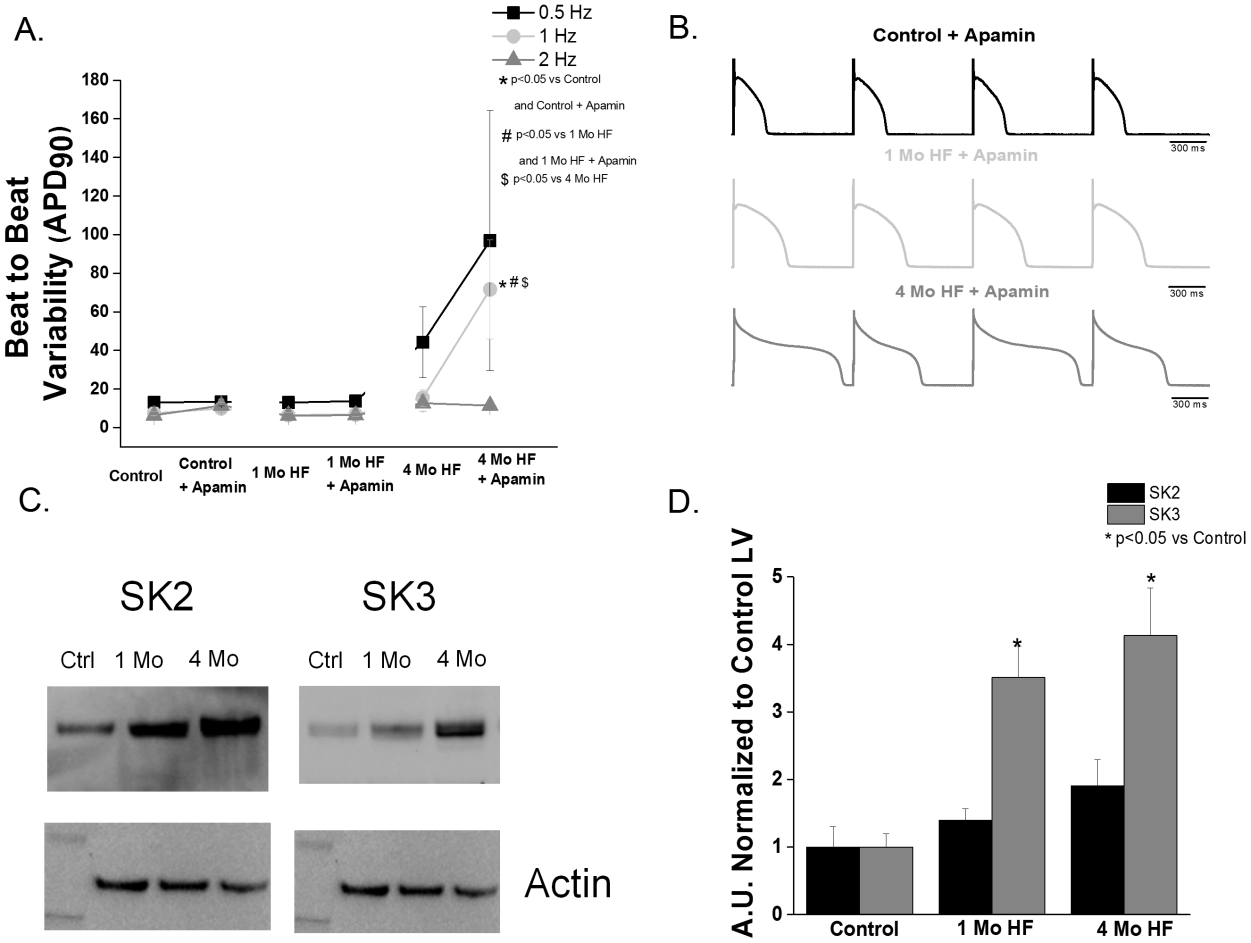
I_Kca_ contributes to ventricular repolarization stability in canine HF, and HF increases SK3 expression. **A**. Beat to beat variability of APD_90_ (BTBV, ms) is unchanged in both 1 month or 4 month HF vs. controls. I_KCa_ block increases the BTBV in the 4 month, but not the 1 month HF group (p<0.05 vs control, 1 month HF and 4 month HF; 2–8 animals per group). **B**. Representative AP tracings of control, 1 month HF and 4 month HF during superfusion with 100 nm apamin. **C**. Representative blots of SK2 and SK3. **D**. SK3 in the 1 and 4 month HF groups is increased (p<0.05 vs control) while SK2 is unchanged (N = 4–5).

In order to assess repolarization instability induced by block of I_KCa_, we measured the beat to beat variability (BTBV) of APD_90_± apamin. HF alone did not increase the BTBV in either HF group compared to controls. Block of I_KCa_ significantly increased BTBV in the 4 month, but not one month, HF group. (Figure 4A,B).

Canine cardiac I_KCa_ encoding proteins SK2 and SK3 were measured in control, 1 month HF and 4 month HF ventricular tissues. No significant change in SK2 protein expression in either HF group (p>0.05 vs control) was found. An ∼4-fold increase in SK3 expression was found in both 1 month and 4 month HF groups. (p<0.05 vs control) (Figure 4C,D).

#### End-stage human heart failure

**Figure 5:**
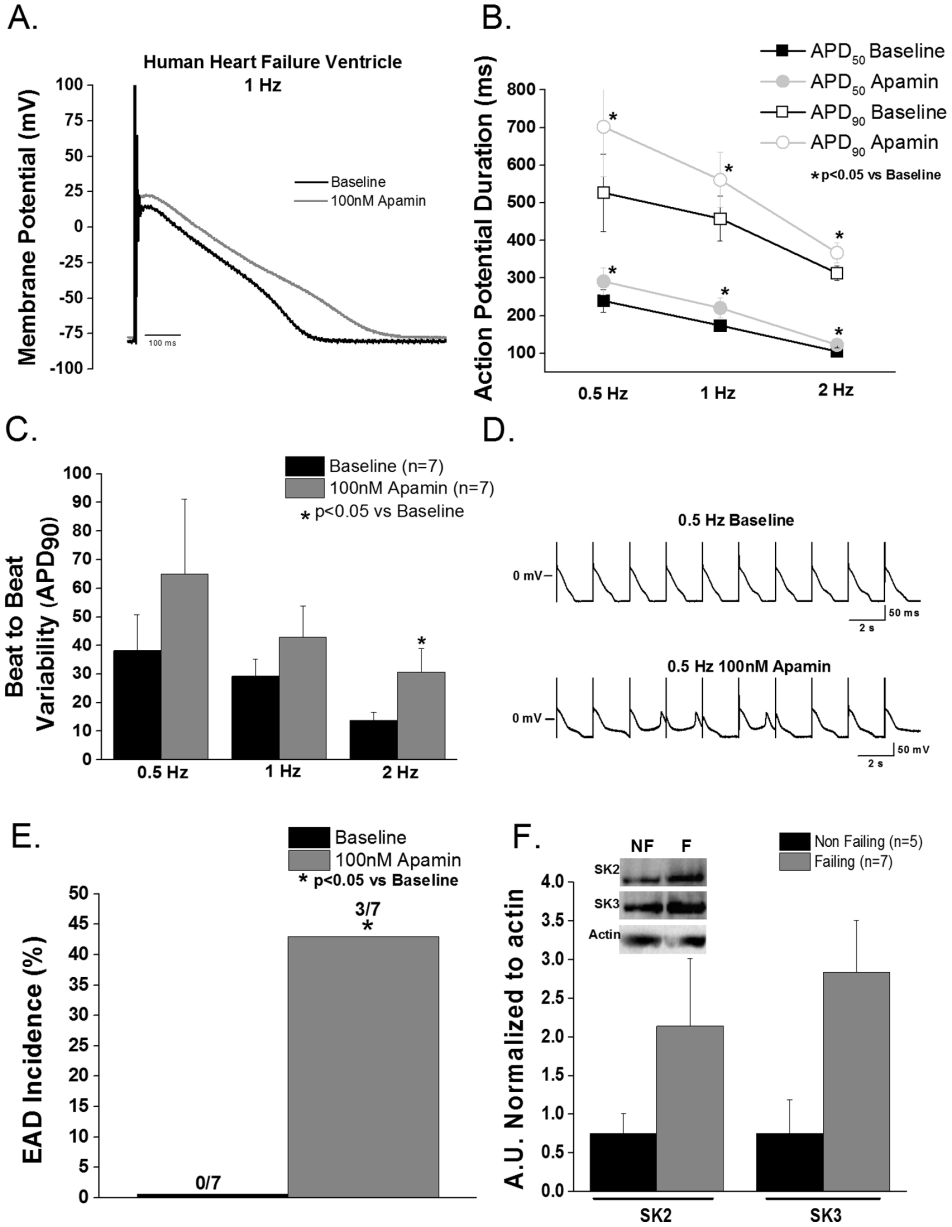
Apamin modulates ventricular repolarization in end-stage human HF. **A**. Representative action potential recorded at 1 Hz from an end-stage human HF ventricular myocyte before and after apamin. **B**. Apamin superfusion prolongs APD_50_ and APD_90_ in end-stage human HF ventricular myocytes at all rates (p<0.05 vs baseline, n = 7). **C**. Apamin superfusion increases (p<0.05 vs baseline) BTBV (ms) at 2 Hz. **D**. Representative action potential showing late phase 3 EADs after apamin superfusion. **E**. Apamin treatment increases EAD incidence in failing human ventricular myocytes. (p<0.05 vs baseline) **F**. Representative blots of control and end-stage human HF SK2 and SK3 proteins (SK2 p = 0.556 and SK3 p = 0.141 vs. control). HF: N = 7 (4 male/3 female); age = 52±13 years and LV ejection fraction of 14.5±5.2%; non-failing controls: n = 5 (2 male/3 female); age = 47±12 years.

In end-stage human HF, inhibition of I_KCa_ (100 nM apamin) caused a significant prolongation of both APD_50_ and APD_90_ compared to baseline (p<0.05, Figure 5 A, B). In addition to AP prolongation, BTBV was significantly increased at 2 Hz in end-stage human HF ventricular cells compared to baseline (Figure 5C). I_KCa_ blockade induced late phase 3 early afterdepolarizations (EADs) in ∼40% of myocytes; while no EADs were observed at baseline (Figure 5D,E).

Human cardiac I_KCa_ encoding proteins SK2 and SK3 were measured in control and end-stage human HF ventricular tissue lysate (Figure 5F). While there was a trend toward increased SK2 expression in HF, this did not achieve statistical significance. SK3 protein also showed a tendency to increase in human end-stage HF compared to control (p = 0.14).

### I_KCa_ inhibition and SK expression in atrial myocytes

#### Canine

**Figure 6:**
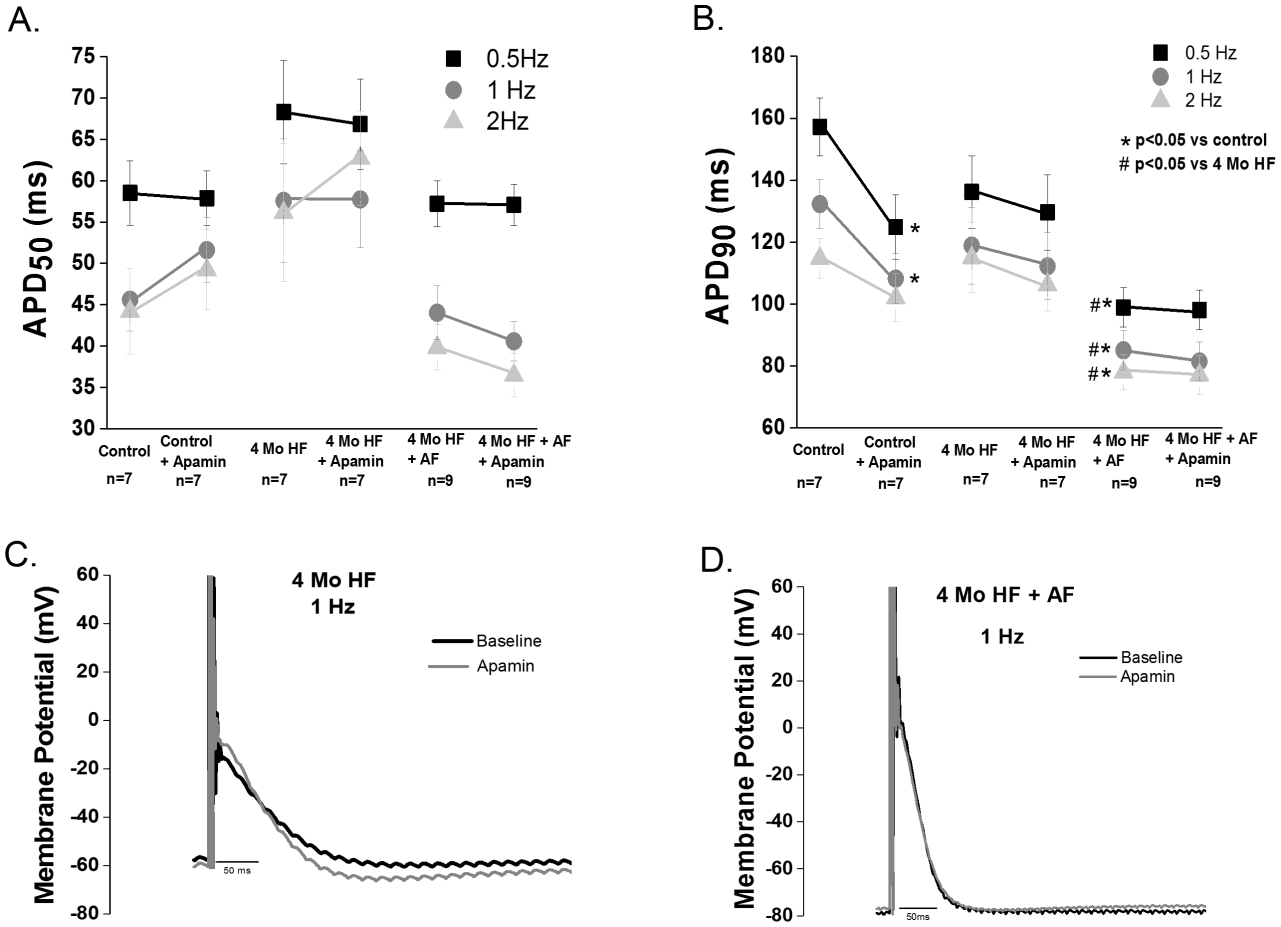
I_KCa_ block does not affect repolarization in normal or diseased atrial myocytes. **A**.100 nM apamin does not affect atrial APD_50_ in any of the studied groups (i.e. control, 4 month HF and 4 month HF+AF, n = 7–9 cells per group) **B**. 100 nM apamin shortened the APD_90_ in controls at 0.5 and 1 Hz. (p<0.05). HF+AF had a shorter baseline APD_90_ compared to control and HF (p<0.05), however no change in APD_90_ was observed after apamin treatment in either 4 month HF or 4 month HF+AF groups. (n = 7–9 cells per groups) **C and D**. Atrial action potential tracings before and after apamin treatment from the 4 month HF group and the 4 month HF+ AF group (2–6 animals per group).

Neither HF nor AF caused any change in APD_50_ compared to control. HF with superimposed AF caused significant APD_90_ shortening compared to control and 4 month HF (p<0.05 vs control and 4 Mo HF). I_KCa_ blockade (100 nM apamin) in control atrial myocytes did not change APD_50_ (Figure 6A) but caused an unexpected shortening of the APD_90_ at 0.5 and 1 Hz (p<0.05 vs baseline) as shown in Figure 6B. I_KCa_ blockade in the 4 month HF and 4 month HF+AF atrial cells did not cause any change in the APD_50_ or the APD_90_ (Figure 6). In further contrast to what we observed in the ventricle, no change in the BTBV of repolarization was observed in either the control, 4 month HF or the 4 month HF+AF groups after I_KCa_ blockade (data not shown). Thus, contrary to what we observed in the ventricle, I_KCa_ does not modulate repolarization in the atria in our chronic HF model.

**Figure 7:**
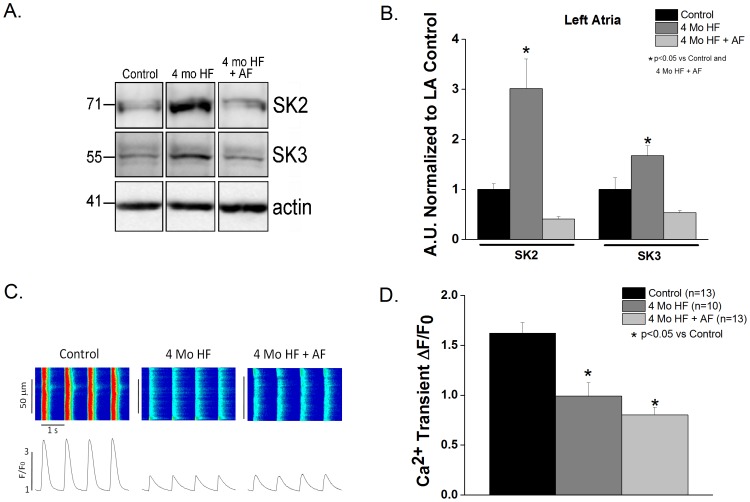
Atrial SK expression and calcium transients in chronic HF with and without AF. **A**. Representative Western blots of SK2 and SK3. **B**. SK2 and SK3 are increased 3- and 2-fold, respectively in the 4 month HF atria. (p<0.05 vs control and 4 month HF+AF) No differences between control and 4 month HF+AF were found in any of the subunits. (N = 3) **C.** Representative calcium transient line scans. **D**. Calcium transient amplitude was decreased in the 4 month HF and 4 month HF+AF groups compared to control (p<0.05 vs control; 3–8 animals per group).

The cardiac I_KCa_ encoding proteins SK2 and SK3 were measured in left atrial appendage tissue from the three groups (i.e. Control, 4 month HF and 4 month HF+AF). A 3-fold and 2-fold increase in SK2 and SK3, respectively in the 4 month HF group was observed compared to both control and 4 month HF+AF groups (p<0.05, Figure 7A, B); while the 4 month HF+AF group did not differ from control. HF, with or without sustained AF, caused a similar significant decrease in calcium transient amplitude compared to controls (p<0.05 vs control, Figure 7C, D).

#### End-stage human heart failure

**Figure 8:**
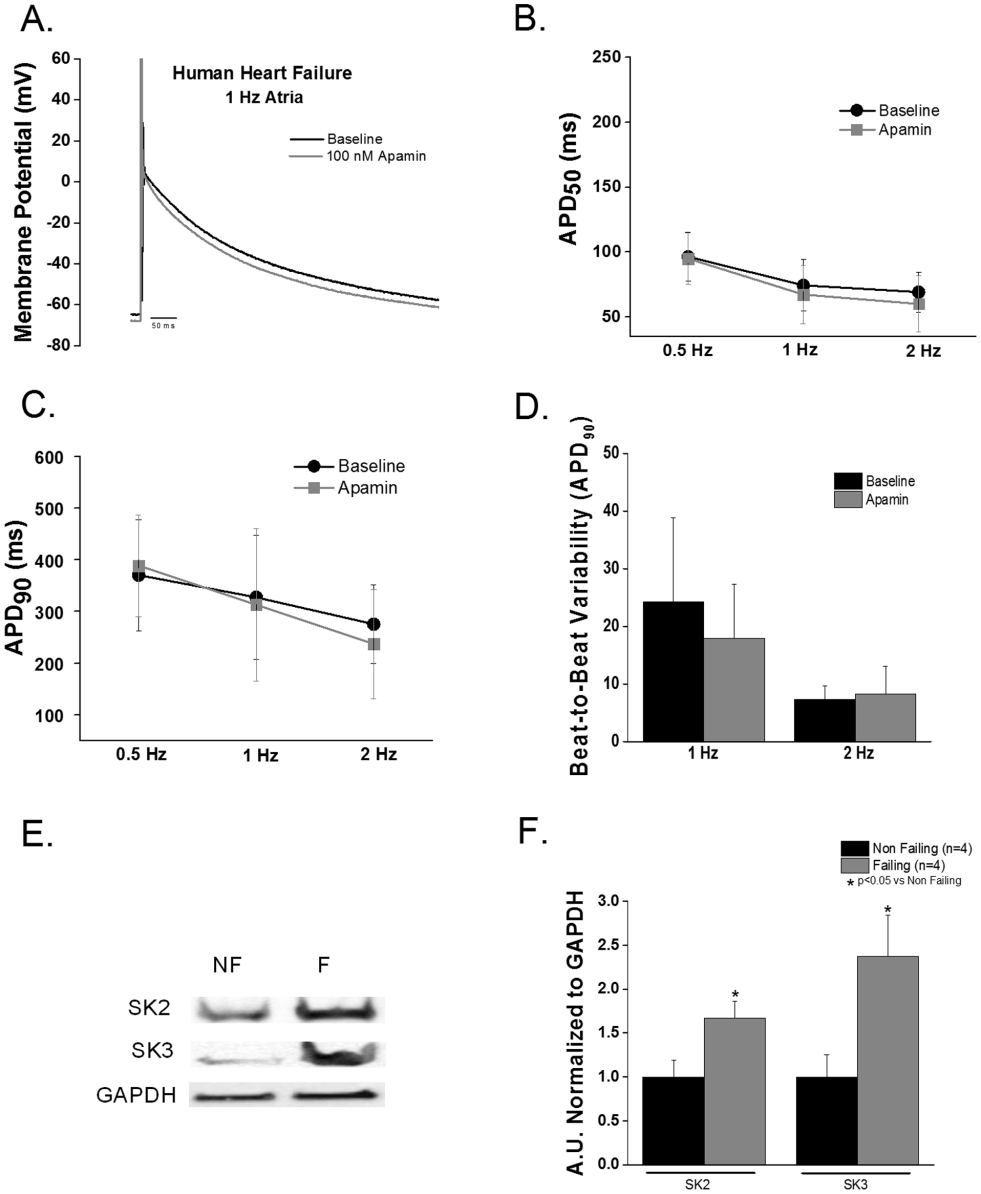
Apamin does not modulate repolarization in end-stage human HF atrial myocytes. **A.** Representative atrial action potential tracing recorded at 1 Hz. **B and C**. Apamin did not change APD_50_ or APD_90_ in human atrial myocytes. (n = 3) **D**. Apamin superfusion did not increase BTBV (ms). **E**. Representative SK2 and SK3 Western blots. **F**. HF increased SK2 and SK3 expression in left atrial tissue (p<0.05 vs non-failing). HF: N = 4 (2 male/2 female); age = 56±4 years and LV ejection fraction of 14.5±1.1%; non-failing controls: n = 4 (2 male/2 female); age = 46±14 years.

Human end-stage HF atrial myocytes showed no significant change in either APD_50_ or APD_90_ when treated with apamin (100 nM). Contrary to what we observed in the ventricular cells no difference was observed in BTBV or afterdepolarizations after apamin treatment in human HF atrial cells (Figure 8). SK2 and SK3 were significantly increased in atrial human HF samples compared with atrial samples from non-failing individuals (p<0.05, Figure 8).

## Discussion

It is well known that HF is a substrate for AF and these are common co-existing disease states. [1], [20] HF patients are at an increased risk for both atrial and ventricular arrhythmias, which contribute to morbidity and mortality. [2] Our main findings were two-fold: first, we did not find any modulation of atrial myocyte repolarization by I_KCa_ in the settings of normal, failing or sustained AF hearts. Secondly, I_KCa_ is activated during HF contributing to stability of ventricular repolarization. Thus, block of I_KCa_ in chronic HF ventricular myocytes prolonged repolarization and increased repolarization instability; these effects have been shown to predict proarrhythmia. [29] Consistent with our findings, I_KCa_ has been previously suggested to play a protective role in the human ventricle during HF. [14].

One interesting question is how I_KCa_ becomes an important modulator of ventricular repolarization during heart failure. Potential explanations for this finding include 1) increased channel expression; 2) altered channel sensitivity to calcium; 3) increased calcium concentrations; or 4) loss of other repolarizing current(s), thereby unmasking the role of I_KCa._

In considering these possibilities, we observed an increase in SK3, but not SK2 in our canine HF model. However, we did not observe a statistically significant increase in either SK2 or SK3 in human HF, although there was a trend toward an increase in SK3 (p = 0.14); our findings are in contrast to a previous report where SK2 expression was increased in human HF. [14] While the expression was not significantly increased in human HF, the inter-species differences we observed may be explained by the intrinsic enhanced variability in explanted human end-stage heart failure samples resulting from inhomogeneities in etiology, comorbidities and drug treatments.

Other possible explanations for our findings are altered channel sensitivity to calcium, altered calcium cycling, or altered repolarization. Recently it was reported in human end-stage HF that SK channel sensitivity to calcium was increased in ventricular myocytes, [14] which could contribute in part to our findings. Of note, other proteins such as: protein kinase, calmodulin and protein phosphatase A, [7], [30] are also known to contribute to the regulation of SK channels, and thus may modulate I_KCa_ during HF.

Since I_KCa_ is a calcium-activated potassium current, HF-induced changes in ventricular calcium handling should directly affect the current. We have previously reported that in our HF model, there is a significant reduction in SR calcium release and calcium transient amplitude, which would reduce rather than augment I_KCa_. [31] In support of this interpretation, a recent report indicates that SR release is necessary and sufficient for I_KCa_ activation. [32] Considering the HF-induced reduction in calcium cycling, and the lack of apamin effect in control cells where calcium cycling is robust, this suggests that altered calcium cycling is not responsible for the protective role of I_KCa_ in heart failure.

Reduced ventricular repolarization reserve may unmask the role of small currents such as I_KCa_. [33] Decreased repolarization reserve is well-described in the ventricle during HF and attributed to reductions in repolarizing currents such as I_K1_, I_Kr_ and I_Ks_. These changes predispose to repolarization instability and/or arrhythmias.[33]–[35] Since I_KCa_ blockade prolonged the AP only during HF and not in controls, we suggest that the contribution of I_KCa_ becomes evident only in settings of decreased repolarization reserve. Thus we suggest that increased channel expression, altered calcium sensitivity of SK channels, or altered repolarization reserve may contribute to the stabilizing role of I_KCa_.

I_KCa_ has been suggested as a therapeutic target for AF. [36] I_KCa_ is defined pharmacologically as apamin-sensitive current, as apamin blocks SK1, SK2 and SK3-encoded channels [9]. One potential problem with this approach is non-selective effects on other ion currents. However, a recent paper surveying apamin effects on human ion channel protein function has demonstrated a high degree of specificity for SK-encoded I_KCa_, even at a concentration five-fold higher than in the present study. [10] A potential limitation of previous studies evaluating I_KCa_ blockade has been a focus on primarily one cardiac chamber; this is limiting since electrical remodeling during HF is chamber-dependent. Specifically during chronic HF, the atrial action potential is shortened [20], [37], [38] while the ventricular action potential is prolonged. [39].

Interest in I_KCa_ as a therapeutic target for atrial arrhythmias followed reports of a genetic predisposition to lone AF attributed to a single nucleotide mutation in the gene KCNN3, which encodes for SK3. [40], [41] The exact mechanism(s) by which a single mutation affects SK channel function remains unclear. Data supporting both loss of function and gain of function as possible mechanisms for AF have been reported in multiple models. [15], [16] Additionally, SK2 and SK3 down regulation have been associated with human AF. [42].

One goal of this study was to elucidate the role of I_KCa_ in atrial electrophysiology during HF and HF with superimposed AF. Considering HF alone, contrary to previous reports, [15], [16] I_KCa_ blockade failed to prolong the atrial action potential in either control or HF atrial myocytes, at physiologic rates. Our findings are different from a recent report where I_KCa_ blockade prolonged the atrial action potential in a whole atrial preparation in a reverse rate-dependent fashion; however this only occurred at rates slower than those used in the present study. [16].

The atrial action potential was not prolonged with I_KCa_ block in HF despite increased both SK2 and SK3 expression. Possible explanations include altered protein trafficking, altered channel calcium sensitivity or altered myocyte calcium handling. We previously reported that HF causes a decrease in calcium current in our 4 months HF tachypacing induced canine model, [20], [43] and in the present study we report reduced calcium transient amplitude. Surprisingly, even in control myocytes where the calcium transient and current are normal, apamin failed to prolong the action potential.

Since a role for I_KCa_ blockers in the treatment of AF has been suggested [11], [36] we also evaluated a HF model with superimposed AF. In a recent report in a canine atrial tachypacing AF model, with preserved LV function, I_KCa_ reduction via a drug which reduced calcium sensitivity of the channel caused a significant prolongation of left atrial action potentials. [15] This contrasts with our AF results in the setting of chronic HF, where I_KCa_ blockade failed to prolong the action potential. Notably, we observed that atrial HF myocytes had similar calcium transient amplitudes whether or not AF was superimposed, suggesting that calcium cycling in HF may be insufficient to activate the current.

In agreement with a previous study of patients with chronic AF who had decreased expression of SK proteins, we found that AF superimposed on HF caused a decrease in the SK2 and SK3 protein expression relative to HF alone. [42] Thus, the lack of apamin effect in the 4 months HF+AF atrial cells may be explained by a decrease in protein expression and/or a decrease in the calcium available for current activation. Since I_KCa_ is a very small current (∼14 pS) [44] and repolarization is accelerated in AF (AP is shortened [45]), it may be less likely that a change in I_KCa_ would affect the overall AP duration. The same logic might apply in chronic HF, where atrial repolarization is also accelerated. [20], [37].

While we did not find a beneficial role for I_KCa_ block in HF or AF, I_KCa_ blockade might have utility in disease states where atrial repolarization is prolonged, or if there is spatial dispersion of atrial repolarization. Additionally, a recent study shows that I_KCa_ blockade in pulmonary veins terminates AF suggesting a potential role for I_KCa_ blockers in paroxysmal AF. [15].

## Limitations

Several studies have shown a gradient of SK channel expression and I_KCa_ current density across the human ventricular wall. [14] However, our experiments used only mid-myocardial myocytes. A similar limitation occurred in the atria, where we only studied cells from the left atrial appendage, and there may be a difference in I_KCa_ between free wall and appendage. [16] Additionally, we used only single cells which may differ in response compared to coupled cells or intact tissue.

One confounding variable in studying I_KCa_ is that the activity varies during the cardiac cycle in a calcium concentration-dependent manner. [32] To assess the role of I_KCa_ in an integrated system, we used perforated patch action potential recordings to permit maintenance of intrinsic calcium cycling, rather than conducting voltage clamp studies to assess the current.

We relied on a pharmacologic approach to define I_KCa_. As with any pharmacologic approach there is a concern about non-specific effects. A recent report evaluated apamin selectivity in multiple human cardiac ion channels including L-type calcium channels, and confirmed the selectivity of apamin for I_KCa_ at 500 nM which is 5-fold higher than the concentration in the present study. [10] However, it has also been reported that apamin inhibits calcium current in neonatal chick and fetal cells. [46] These reported differences of apamin on calcium current may reflect maturation-dependent differences in channel subunit expression. [47] We did not directly evaluate the effects of apamin on calcium current, but our experimental system is closest to that in the recent report by Yu et al. [10], suggesting that block of calcium current was unlikely to mediate the observed effects of apamin in the present study.

## Conclusions

These experiments highlight the need to evaluate novel therapeutic targets for arrhythmias in both atria and ventricles. In chronic HF, I_KCa_ plays a protective role in the ventricle and currrent block is proarrhythmic. Notably, in early HF (1 month canine HF), I_KCa_ blockade is not proarrhythmic, perhaps reflecting a relatively preserved repolarization reserve with a diminished dependence on I_KCa_ for repolarization stability compared to chronic HF.

We found that I_KCa_ does not play a role in repolarization in the atria as current block does not prolong the action potential in either human or canine HF. Similarly, I_KCa_ does not play a role in repolarization of the atrial AP during sustained AF with concurrent chronic HF, despite increased protein expression.

Collectively, our data do not support a role for I_KCa_ blockers for the treatment of atrial arrhythmias. Rather, our findings suggest that therapeutic strategies to reduce I_KCa_ may be unsafe in the setting of atrial arrhythmias with concurrent HF due to potential proarrhythmic effects in the ventricles.
